# Photoluminescence Enhancement of Adsorbed Species on Si Nanoparticles

**DOI:** 10.1186/s11671-015-1220-9

**Published:** 2016-01-07

**Authors:** Taketoshi Matsumoto, Masanori Maeda, Hikaru Kobayashi

**Affiliations:** The Institute of Scientific and Industrial Research, Osaka University , 8-1, Mihogaoka, Ibaraki, Osaka 567-0047 Japan

**Keywords:** Si nanoparticle, Si swarf, Photoluminescence, Absorbance, Enhancement, Anthracene

## Abstract

We have fabricated Si nanoparticles from Si swarf using the beads milling method. The mode diameter of produced Si nanoparticles was between 4.8 and 5.2 nm. Si nanoparticles in hexane show photoluminescence (PL) spectra with peaks at 2.56, 2.73, 2.91, and 3.09 eV. The peaked PL spectra are attributed to the vibronic structure of adsorbed dimethylanthracene (DMA) impurity in hexane. The PL intensity of hexane with DMA increases by ~3000 times by adsorption on Si nanoparticles. The PL enhancement results from an increase in absorption probability of incident light by DMA caused by adsorption on the surface of Si nanoparticles.

## Background

Si nanoparticles possess significant properties such as wide-bandgap energies due to the quantum confinement effect [1, 2], large surface area [3], and high reactivity [4]. Moreover, Si is a safe material, possible to apply to the bio-medical field [5]. Extensive studies have been performed to produce Si nanoparticles using various methods including laser ablation [6, 7], plasma-enhanced chemical vapor deposition (CVD) [8], hot wire CVD [9], sputtering [10], Si implantation [11], intense pulsed ion beam evaporation [12], and co-evaporation of SiO plus SiO_2_ with subsequent heat treatment [13]. These methods require vacuum chambers and are time- and cost-consuming. Si nanoparticles can also be produced by chemical methods such as Zintl reaction and LiAlH reduction of SiCl_4_ [14]. For a cheap fabrication method of Si nanoparticles, Lam et al. [15] employed the ball milling method, but only a small fraction of Si powders becomes nanometer size. We have developed a fabrication method of Si nanoparticles from Si swarf (i.e., industrial waste produced during slicing Si ingots) by use of the beads milling method [16, 17].

Si nanoparticles emit visible light under UV irradiation, applicable to light-emitting diodes [18] and biomarkers [5]. Kang et al. [14] have shown that various color photoluminescence (PL) can be obtained from Si nanoparticles by varying the size of Si nanoparticles by oxidation.

PL from Si nanoparticles is differently attributed to several causes. In cases where the PL energy strongly depends on the size of Si nanoparticles, it arises most likely from band-to-band transition of Si nanoparticles because the bandgap energy is determined by its size due to the quantum confinement effect [1, 2]. On the other hand, Zhu et al. [19] and Inada et al. [20] have attributed PL from Si nanoparticles to energy states of SiO_2_ (most probably interface states) formed on Si nanoparticles, and in this case, the PL energy does not depend on the size of Si nanoparticles. It is also highly probable that PL is influenced by adsorbed species on Si nanoparticles. Adsorption of alkyl groups on Si nanoparticles is reported to cause blue shift due to prevention of transfer of electron-hole pairs from small (i.e., wide bandgap) to large (i.e., narrow bandgap) nanoparticles [21]. Complicated structured PL spectra, e.g., three-peaked structure, have been observed for Si nanoparticles in hexane, which may arise from adsorbed species [22–24].

In the present study, Si nanoparticles have been fabricated from Si swarf by use of the beads milling method. Si nanoparticles in hexane show PL spectra with four peaks which are attributable to the vibronic structure of 9,10-dimethylanthracene (DMA) impurity in hexane. The PL intensity is increased by ~3000 times by adsorption of DMA on Si nanoparticles, and it is attributed to enhancement of excitation probability of DMA by adsorption on Si nanoparticles.

## Methods

After cleaning Si swarf, Si nanoparticles were produced by use of the ball milling and beads milling methods. In the case of one-step milling, 0.5-mm zirconia beads were employed. For two-step milling, milling with 0.5-mm zirconia beads and then with 0.3-mm zirconia beads was performed in 2-propanol. Fabricated Si nanoparticles were filtered by use of a Teflon membrane filter, followed by immersion in hexane plus water. In some cases, 2 ml mixed solutions of 70 wt% HNO_3_ plus 50 wt% HF (HNO_3_:HF = 1:1 in volume) were added to 5 ml hexane containing Si nanoparticles.

X-ray diffraction (XRD) measurements were performed using a RIGAKU SmartLab diffractometer. Transmission electron microscopy (TEM) micrographs were observed using a JEOL JEM-3000F microscope at an incident electron energy of 300 keV. Measurements of PL spectra were carried out with a JASCO FP-8500 spectrometer after removal of aggregated and precipitated Si nanoparticles. Ultraviolet and visible light (UV-Vis) absorption spectra were recorded using a JASCO V-670 spectrometer. The PL quantum efficiencies were measured by a Hamamatsu Photonics K.K. Quantaurus-QY absolute PL quantum yields measurement system. The absolute PL quantum efficiencies were calculated from the ratio of a PL photon number to an absorbed photon number. These photon numbers were determined from a differential spectrum between two spectra of hexane solutions with and without Si nanoparticles.These spectra were measured with an integrating sphere to collect photons emitted in all directions into a spectrometer. A downward peak in a differential spectrum was due to the increase in absorption of excitation light caused by the addition of Si nanoparticles, while an upward peak was attributable to PL.

## Results and Discussion

**Fig. 1:**
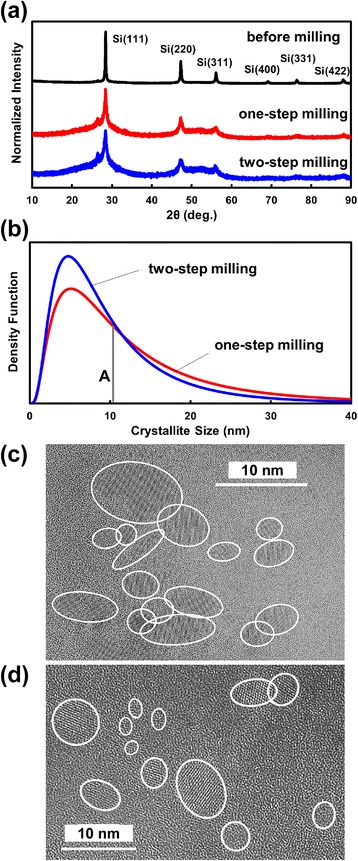
XRD patterns of Si nanoparticles fabricated from Si swarf before and after one-step and two-step beads milling (**a**), and volume distribution of Si nanoparticle diameter fabricated by one-step milling and that of Si nanoparticles fabricated by two-step milling (**b**). TEM micrograph of Si nanoparticles fabricated by one-step (**c**) and two-step milling (**d**)

Figure 1a shows the XRD patterns for Si nanopowders fabricated from Si swarf before and after the beads milling procedure. Before beads milling, all the diffraction peaks were sharp, indicating large crystallite sizes. All the observed peaks were attributable to crystalline Si, and the (111) peak was the most intense. The beads milling procedure did not change the intensity ratio of the diffraction peaks, while all the peaks were considerably broadened. The widths of the XRD peaks for Si nanoparticles produced by the two-step milling method were larger than those for one-step milling, showing that the two-step milling effectively decreased the size of Si nanoparticles. These results show that the shape of Si powders remained almost unchanged by the beads milling procedures, but the sizes became considerably smaller.

Figure 1b shows the volume distribution of Si nanoparticles vs. the diameter estimated from the analysis of the XRD (111) peak shape, obtained using the SLN profile method [25]. For the one-step milling method, the maximum distribution (i.e., mode diameter) was present at 5.2 nm, the median diameter (cf. vertical line A by which the total area is divided into the two same areas) was 10.5 nm, and the average diameter was 13.2 nm. For the two-step milling method, the mode diameter, the median diameter, and the average diameter were estimated to be 4.8, 8.4, and 10.2 nm, respectively.

**Fig. 2:**
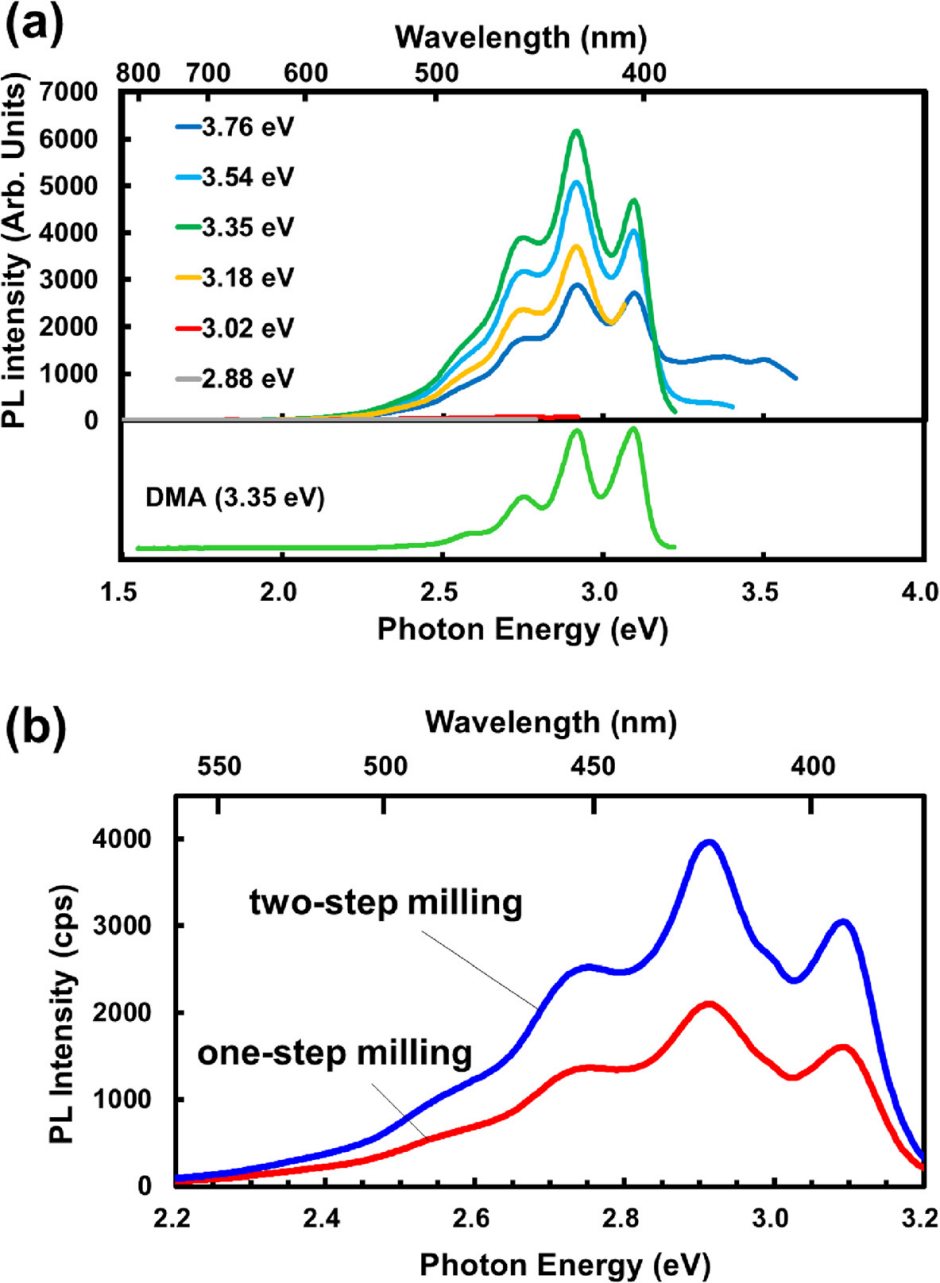
PL spectra of Si nanoparticles fabricated from Si swarf by use of one-step milling measured under various excitation energies (**a**), and PL spectra of Si nanoparticles fabricated using the one-step and two-step beads milling methods (**b**)

Figure 1c, d shows the TEM micrographs of Si nanoparticles fabricated by the one-step and two-step milling methods, respectively. It is clearly seen that most of Si nanoparticles were less than 10 nm, in good agreement with the above-explained Si nanoparticle size distribution. The TEM micrographs clearly show that the average size of Si nanoparticles produced by the two-step milling method (Fig. 2d) was smaller than that of the one-step milled Si nanoparticles (Fig. 2c). This TEM result shows good agreement with the XRD result because the average diameters of Si nanoparticles fabricated by the one-step and two-step milling methods observed in the TEM micrographs were 4.1 and 3.8 nm, respectively. TEM micrographs typically showed that Si nanoparticles were well dispersed as seen in Fig. 1 presumably due to low concentration and no ordering.

Figure 2 shows the PL spectra for Si nanoparticles fabricated from Si swarf by use of the beads milling method in hexane. The PL spectra had a peaked structure with peaks at 2.56, 2.73, 2.91, and 3.09 eV. The PL spectra hardly depended on the incident light excitation energy of 3.18–3.76 eV (Fig. 2a). If PL arose from the band-to-band transition of Si nanoparticles, the PL energy would strongly depend on the excitation energy because with an increase in the excitation energy, wider bandgap Si nanoparticles can be excited, leading to PL emission with higher energy. This is not the case, and the observed PL most probably arises from adsorbed species on Si nanoparticles. The PL spectra of Si nanoparticles were very similar to that of DMA (bottom spectrum in Fig. 2a) with peaks at 2.55, 2.75, 2.92, and 3.09 eV. The peaked structure with almost the identical energy separation of ~0.18 eV is due to the vibronic structure of DMA molecules [26]. We conclude that the observed PL with the vibronic structure resulted from DMA impurity in hexane adsorbed on Si nanoparticles.

Figure 2b compares the PL spectra of Si nanoparticles produced by the one-step and two-step milling methods. Both the spectra possessed a peaked structure with nearly the same peak energies. In the case of two-step milling, the PL intensity was approximately twice that for one-step milling.

The same experiment was carried out using Si nanoparticles in 2,2,4-trimethyl pentane (TMP) instead of hexane, but no PL peaks attributable to DMA were observed probably because TMP did not contain DMA. It was also confirmed that PL intensity for vibronic structures increased by the addition of a 2 × 10^−8^ M DMA solution in hexane. These results clearly show that the vibronic structure arises from DMA molecules adsorbed on Si nanoparticles, and the PL intensity is enhanced by Si nanoparticles.

**Fig. 3:**
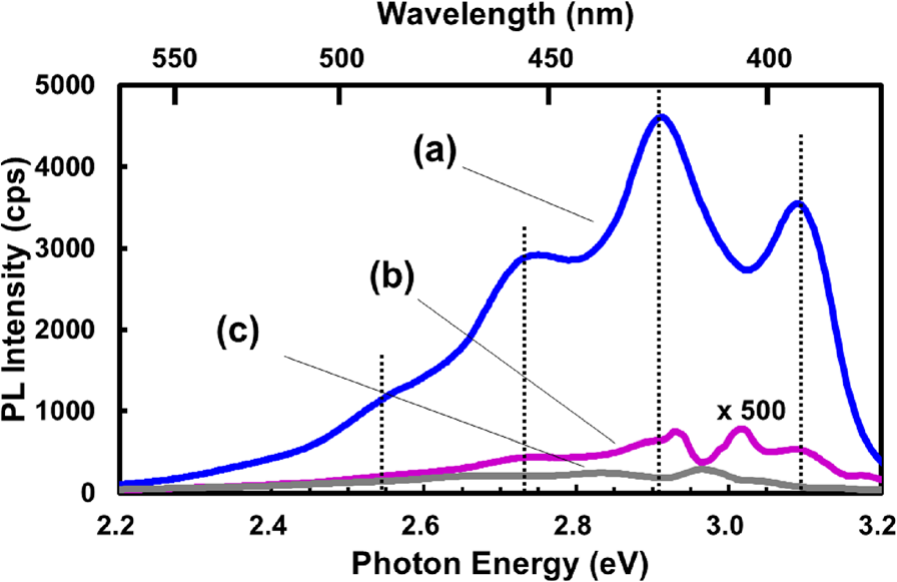
PL spectra: (*a*) for Si nanoparticles in hexane, (*b*) for hexane without Si nanoparticles, (*c*) after addition of HNO_3_ plus HF solutions to specimen (*a*)

Figure 3 shows the PL spectra of Si nanoparticles in hexane with (spectrum c) and without (spectrum a) HF plus HNO_3_. It is well known that HF plus HNO_3_ solutions dissolve Si by oxidation with HNO_3_ to form SiO_2_ and etching of SiO_2_ by HF [27]. By Si dissolution, the PL intensity decreased to ~1/13 while the PL peak positions were nearly unchanged. By Si dissolution, adsorbed DMA dissolves in the solution. This result demonstrates that the PL intensity is enhanced by the presence of Si nanoparticles. Even in cases where the concentration of adsorbed DMA is much higher than that in the solution, PL intensity would not change without enhancement because Si nanoparticles are uniformly distributed in the solution, i.e., the PL enhancement cannot be explained by selective adsorption of DMA on Si nanoparticles. The remaining PL intensity with HF plus HNO_3_ is likely to result mainly from undissolved Si nanoparticles most likely chemically stabilized by adsorption of DMA.

Spectrum b in Fig. 3 is for hexane without Si nanoparticles. For this spectrum, impurity in hexane was concentrated to 39 times by heating hexane, and the spectrum for unconcentrated hexane was subtracted from the spectrum for concentrated hexane. The structure of the spectrum in the energy region between 2.8 and 3.1 eV could not be determined because of the presence of a Raman peak with a strong intensity at 2.97 eV. The peaked structure nearly the same as those for Si nanoparticles in hexane was observed (cf. vertival dotted lines), but the PL intensity was much lower. From the ratio in the PL intensity for Si nanoparticles in hexane to that for hexane without Si nanoparticles, the PL enhancement factor was determined to be ~3000. Several researchers have observed PL enhancement by the presence of silver particles, and the enhancement is attributed to the plasmonic effect [28, 29]. The observed PL enhancement without Ag is not attributable to the plasmonic effect but to an increase in absorption probability of incident light by DMA due to adsorption on Si nanoparticles, as explained below.

**Fig. 4:**
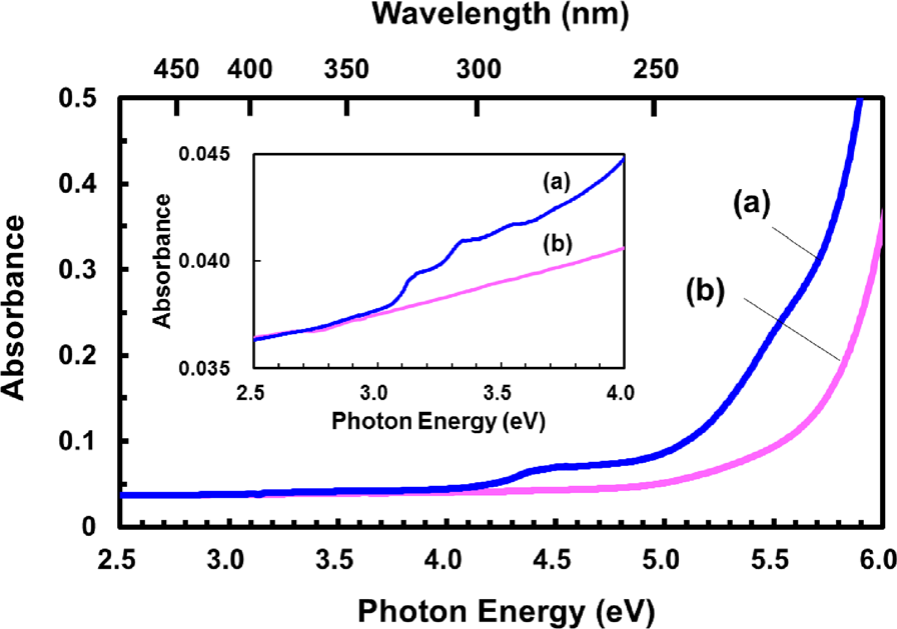
Absorption spectra of hexane with (*a*) and without (*b*) Si nanoparticles. The *inset* shows the same spectra with the expanded vertical axis

Figure 4 compares the UV-Vis absorption spectra of hexane with and without Si nanoparticles. It is clearly seen that the absorbance in the incident light wavelength region between 3.2 and 3.6 eV used for PL measurements increased by the presence of Si nanoparticles. It should be noted that the peaked structure similar to those in the PL spectra was observed in the absorption spectrum. The energy separation of the peaks was 0.19 eV, which corresponded to the vibrational energy of the breathing vibration mode of the excited state. This energy separation was slightly larger than that of the peaks in the PL spectra where the peak separation corresponded to the vibrational energy of the ground state. This corresponds to the slightly shortened C-C bond length in an excited anthracene molecule [30]. The observation of vibronic structure in the absorption spectrum clearly shows that incident light is directly absorbed by adsorbed DMA, but not by Si nanoparticles. Therefore, it can be concluded that excitation probability (light absorption probability) of DMA is greatly (i.e., ~3000 times) increased by adsorption on Si nanoparticles.

**Fig. 5:**
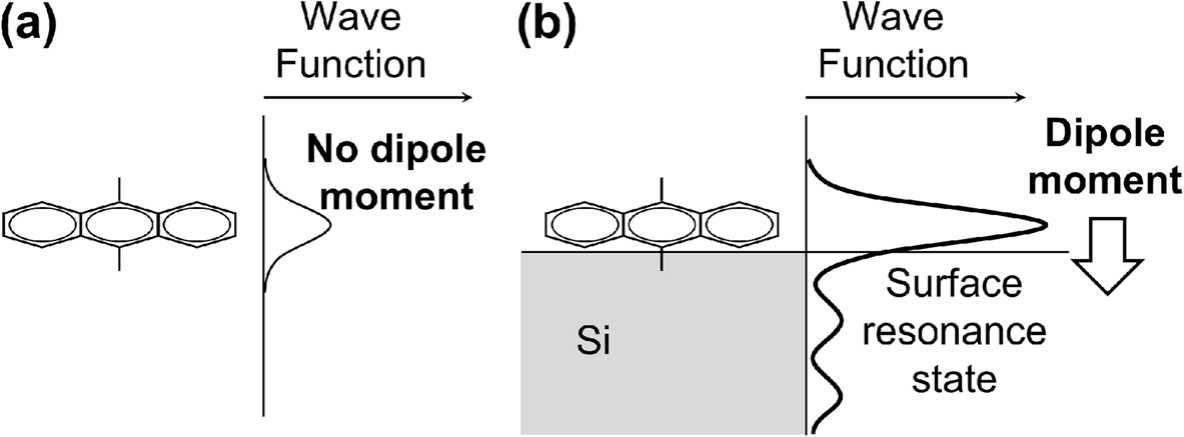
Possible mechanism of enhancement of PL intensity of DMA by the presence of Si nanoparticles in hexane: without a Si nanoparticle (**a**) and with a Si nanoparticle interacting with DMA (**b**)

Figure 5 schematically shows the possible mechanisms for the enhancement of the excitation probability. The excitation probability, *P*_*T*_, is proportional to the square of the transition moment:

1 $$ {P}_T\propto {\left[{\displaystyle \int {\psi}_f*e\boldsymbol{\mathsf{r}}}\ {\psi}_id\tau \right]}^2, $$

where *ψ*_*f*_ and *ψ*_*i*_ are the wave functions of final and initial states, respectively, and *e****r*** is the dipole moment. The following are possible mechanisms to increase the excitation probability (cf. Fig. 5b): (i) dipole moment is induced by charge transfer between DMA and Si, and (ii) wave functions of DMA are enhanced possibly due to formation of surface resonance state [31] by overlapping of the wave functions of DMA and Si. Effects (i) and (ii) increase *e****r*** and *ψ*_*i*_, respectively, in Eq. (1). Both the effects increase the transition moment. More detailed experimental and theoretical studies are needed to clarify the mechanism of the PL enhancement.

The PL intensity of Si nanoparticles produced by the two-step milling method with a mode diameter of 4.8 nm is approximately twice that of Si nanoparticles fabricated by the one-step milling method (mode diameter 5.2 nm). From the mode diameter, the ratio in the surface areas of Si nanoparticles fabricated using the one-step and two-step milling methods is estimated to be 1:1.1, and therefore, the difference in the surface area cannot explain the PL intensity ratio of 1:2. The abovementioned effects, effects (i) and (ii), may be enhanced as the size of Si nanoparticles decreases. The great PL enhancement after DMA adsorption for the two-step milling method is most likely due to smaller Si nanoparticles with a higher concentration of active adsorption sites.

The PL quantum efficiencies can be estimated from measurements of the PL intensity divided by the absorbed light intensity. The absorbed light intensity was estimated from the incident light intensity and the integrated light intensity after passing through Si nanoparticle-containing hexane. The PL quantum efficiencies for Si nanoparticles produced by the one-step and two-step milling methods are estimated to be 9 and 15 %, respectively.

## Conclusions

The PL intensity is enhanced by ~3000 times by adsorption of DMA on Si nanoparticles. The PL and UV-Vis absorption spectra possess peaked structures, which are attributable to vibronic transitions including vibrational energies of DMA in the ground state and the excited state, respectively. The PL enhancement results from a great increase in light absorption probability of DMA by adsorption on the surface of Si nanoparticles. The structure of the PL spectra is independent of the excitation energy and the size of Si nanoparticles, while the PL intensity strongly depends on the size of Si nanoparticles.
